# Development and Evaluation of Fucoidan-Loaded Electrospun Polyvinyl Alcohol/Levan Nanofibers for Wound Dressing Applications

**DOI:** 10.3390/biomimetics9090508

**Published:** 2024-08-23

**Authors:** Natavan Ismayilova, Muhammad Khaqan Zia, Hatice Selen Akkaya, Songul Ulag, Yeliz Guldorum, Ebru Toksoy Oner, Erol Ince, Liviu Duta, Oguzhan Gunduz

**Affiliations:** 1 Chemical Engineering Department, Engineering Faculty, Istanbul University-Cerrahpasa, 34320 Istanbul, Turkey; nataone.chem@gmail.com; 2 Center for Nanotechnology & Biomaterials Application and Research (NBUAM), Marmara University, 34722 Istanbul, Turkey; songul.ulag@marmara.edu.tr (S.U.); ersanyeliz94@gmail.com (Y.G.); 3 Interdisciplinary Research Centre in Biomedical Materials (IRCBM), COMSATS University Islamabad, Lahore Campus, Lahore 54000, Pakistan; khaqanzia786@gmail.com; 4 Biomedical Engineering Department, Rheinisch-Westfälische Technische Hochschule Aachen, Faculty of Medicine, 65428 Aachen, Germany; selenakkaya18@gmail.com; 5 Department of Metallurgical and Materials Engineering, Faculty of Technology, Marmara University, 34722 Istanbul, Turkey; 6 Health Institutes of Türkiye (TUSEB), 34718 Istanbul, Turkey; 7 Department of Biomedical Engineering, Electrical and Electronics Faculty, Yildiz Technical University, 34210 Istanbul, Turkey; 8 IBSB—Industrial Biotechnology and Systems Biology Research Group, Department of Bioengineering, Marmara University, 34722 Istanbul, Turkey; ebru.toksoy@marmara.edu.tr; 9 Lasers Department, National Institute for Laser, Plasma and Radiation Physics, 077125 Magurele, Romania

**Keywords:** electrospinning, Halomonas Levan, fucoidan, polyvinyl alcohol, wound dressing

## Abstract

Wound dressing is an ancient technique for promoting healing, and modern technology has led to the development of advanced dressings that enhance patient care. Nanofiber-based wound dressings are a medical innovation with enhanced properties, including improved adhesion, reduced infection rates, and increased tissue regeneration. This article focuses on electrospun nanofibrous wound dressing materials produced using the widely adopted method of electrospinning. This article explores several parameters that influence fiber size, including electrical conductivity, electric potential, collector distance, viscosity, flow rate, and surface tension. With Fucoidan (FUC) loading, an increase in the fiber diameter of the control group from 310 nm to 395 nm was observed. This research also examines the use of Halomonas Levan (HL), a polysaccharide, and polyvinyl alcohol (PVA) polymer as wound dressing materials to enhance the mechanical properties of the latter. The incorporation of various concentrations of FUC into PVA-HL electrospun nanofibers yielded diverse effects on tensile strength: an enhancement was observed in the PVA-HL-10FUC formulation, while reductions were noted in the PVA-HL-13FUC and PVA-HL-15FUC formulations. The WST1 assay demonstrated that none of the samples exhibited cytotoxicity up to 72 h, as cell viability increased over time. In conclusion, nanofibrous PVA-HL structures loaded with FUC, which promote tissue regeneration and prevent infection, could be considered a novel wound dressing material.

## 1. Introduction

Wound dressing is an ancient healing technique that has been used for thousands of years. Throughout human history, natural materials have played a pivotal role in various applications, exemplified by the use of leaves, honey, spider silk, tree bark, vinegar, beer, and wine [1]. Ancient Egyptians used honey and animal fats to promote wound healing, and in the Middle Ages a dressing made of egg yolks and rose oil was used for wound treatment [2]. Antiseptic wound dressing was first introduced in the 19th century when carbolic acid-soaked fabrics were used to prevent infection [3]. Cotton wool and gauze dressings were used in World War I to prevent infection and absorb wound exudate, while World War II saw the introduction of “sulfa powder dressing” made of sulfa drugs and plaster of Paris. Technological and medical advancements in the 20th century brought about more advanced wound dressings such as hydrogels, foams, and transparent films designed to promote moist wound healing, prevent infection, and speed up healing [4]. Today, wound dressing technology continues to evolve, with new materials and techniques being developed to provide better care for patients with all types of wounds, from minor cuts and scrapes to severe burns and surgical incisions [5]. A proper wound dressing material should provide a certain moisture level to promote tissue regeneration and protect the wound from infection. Along with that, a wound dressing should also remove excess exudate and avoid maceration while maintaining gas exchange [6]. In this work, a nanofibrous polyvinyl alcohol (PVA)–Halomonas Levan (HL) structure loaded with Fucoidan (FUC) was tested as a novel wound dressing material. This nanofiber structure was obtained by electrospinning a dissolved PVA-HL solution.

Nanotechnology involves the design, production, and application of materials at the nanoscale level. It has a wide range of applications, including in medicine, where it has been used to develop innovative wound dressing materials [7]. It has enabled the development of wound dressing materials with enhanced properties, such as improved adhesion, reduced infection rates, and increased tissue regeneration [8]. Nanofiber-based wound dressings, an innovative application of nanotechnology in medicine, have high surface area-to-volume ratios, which enhance their ability to absorb wound exudate and promote tissue regeneration [9]. They also have good mechanical properties and can conform well to irregular wound surfaces. Additionally, nanofiber-based wound dressings can be easily modified to incorporate bioactive agents, such as antimicrobial agents or growth factors, which can further enhance their therapeutic properties [9]. Nanofibers can be produced using various methods, including electrospinning, self-assembly, and phase separation [10]. This work mainly focuses on electrospun nanofibrous wound dressing materials.

Electrospinning is a popular method used to create a fibrous structure from a polymer solution [11]. The technique involves passing the solution through a needle subjected to a voltage, which breaks down intermolecular forces, forming micro/nanostructures [12]. The mechanism behind electrospinning is rooted in the competition between Coulomb and cohesion forces within the droplet. The electric field causes the droplet interface to deform, breaking its cohesive forces and producing droplets that range in size, from micrometers to nanometers. The prevailing Coulomb force releases the surface tension, forming nanodroplets [13]. Electrospinning only requires a four-part setup: a high-voltage power supply, a pump to control the flow rate, a flat-tip needle for the solution to pass through, and a conducting collector, which is where the fibrous material is collected [14]. In this study, a PVA-HL-based aqueous solution was passed through a high-voltage applied needle and a nanofibrous structure was obtained.

HL is a type of polysaccharide that consists of fructose units linked together through β-(2→6) glycosidic bonds [15]. Its molecular structure is a linear polymer that has a branched structure, with a degree of polymerization that can range from 3 to 5000 units [16]. HL, a sugar-based polymer with unique properties conducive to wound healing and tissue engineering applications, stands out for its excellent biocompatibility and biodegradability, which render it suitable for medical applications. Its adhesive nature, coupled with its anti-inflammatory and antibacterial properties, can foster tissue regeneration, and mitigate the risk of infection [17,18]. However, its limited availability, processing difficulties, and lack of standardization limit its commercial use. Additionally, there is a lack of research on its use in these applications, which has hindered its widespread adoption [19]. In this study, HL is used together with PVA polymer with the aim of improving the mechanical properties of the latter.

PVA is a water-soluble synthetic polymer that is produced by the hydrolysis of polyvinyl acetate. The molecular structure of PVA consists of a long chain of repeating units of vinyl alcohol, which is a monomer formed by the hydrolysis of vinyl acetate [20]. Each vinyl alcohol unit contains a hydroxyl (–OH) group and a vinyl group (–CH=CH_2_). PVA can form hydrogen bonds with water molecules due to the (–OH) groups present in the PVA chains [21].

To improve its water resistance, PVA can be crosslinked through various methods, such as chemical crosslinking or physical crosslinking. Crosslinking improves the mechanical properties and water resistance of PVA by forming a network of chemical or physical bonds between its polymer chains [22]. Crosslinked PVA has a more three-dimensional structure than non-crosslinked PVA. This makes it more resistant to deformation and dissolution in water. It also has improved mechanical properties such as increased stiffness, strength, and thermal stability compared to non-crosslinked PVA [23]. The chemical crosslinking of PVA involves the use of chemical crosslinkers that react with the (–OH) groups present in the PVA chains. The most common chemical crosslinkers used for PVA are aldehydes, such as formaldehyde, glutaraldehyde, and glyoxal [24].

PVA’s biocompatibility, non-toxicity, and water solubility also make it a versatile material for tissue engineering applications. In wound dressing applications, PVA nanofiber mats can be used to cover a wound and provide a moist environment for wound healing. The high surface area-to-volume ratio of PVA nanofibers allows for the increased absorption and retention of wound exudates, which can promote wound healing. PVA nanofiber mats can also be functionalized with bioactive molecules, such as growth factors and antibiotics, to enhance the healing process and prevent infections [25,26].

A PVA-HL mixture solution is expected to generate improved wound healing properties due to the combination of PVA’s film-forming ability and barrier properties and HL’s water-holding and emulsifying properties [27]. The resulting solution is expected to be biocompatible, making it suitable for biomedical applications. However, there might be compatibility issues due to the chemical differences between the two polymers. Also, PVA has limited solubility in water at high concentrations and a low pH, while Levan is only soluble in water in a narrow range of pH and temperatures. Mixing these two polymers in a solution can result in limited solubility, making it sometimes difficult to handle and process. One should mention that there are a few approaches that could be used to solve these problems, such as (i) solvent selection an optimization (i.e., the identification of a common solvent or a solvent system, optimum solvent ratios, the proper adjustment of the temperature), (ii) pH adjustment, (iii) ultrasonication, (iv) polymer modification (i.e., chemical modification, blending additives), (v) solution preparation techniques (i.e., sequential dissolution, controlled mixing), (vi) emulsion techniques, (vii) the use of crosslinkers, or (viii) thermal treatments (i.e., annealing).

FUC is a type of sulfated polysaccharide found in various species of brown seaweed [28]. The antimicrobial activity of FUC is attributed to its ability to interact with the cell walls of microorganisms, disrupting their structural integrity and inhibiting their growth and proliferation [29]. When FUC is incorporated into a nanofibrous wound dressing, it can provide several benefits. Firstly, it can help prevent infection by inhibiting the growth of bacteria and other microorganisms in the wound area. Secondly, it can promote wound healing by stimulating the proliferation and migration of the cells involved in the wound-healing process. Additionally, FUC has been shown to have anti-inflammatory and antioxidant properties, which can further promote wound healing and reduce scarring [30].

In this research, a novel material for wound dressing was thoroughly examined. The method used to obtain the wound dressing involved passing an aqueous solution of HL polysaccharide and PVA synthetic polymer through a high-voltage applied needle, resulting in the production of a nanofibrous electrospun mat. FUC, a potent antibiotic agent, was utilized in the wound dressing material. The application of HL polysaccharide served two purposes: to boost cell proliferation and to enhance the mechanical properties of the PVA nanofibers. Comprehensive physical–chemical investigations were performed on the resulting structures, which included measuring their tensile strength, thickness, and morphological properties (by scanning electron microscopy, SEM, Zeiss, Oberkochen, Germany), chemical (via Fourier-transform infrared spectroscopy, FTIR) and thermal analyses (via differential scanning calorimetry testing, DSC, Shimadzu, Tokyo, Japan). Furthermore, the in vitro biocompatibility and drug release tests of the wound dressing material were also investigated.

The intersection of biomimetic design and electrospinning has led to significant innovations in the biomedical field, particularly in tissue engineering and regenerative medicine. Researchers are increasingly using the capabilities of electrospinning to create scaffolds that closely mimic both the structure and function of the extracellular matrix (ECM). These electrospun scaffolds are designed to provide a supportive framework for cell attachment, proliferation, and differentiation, thereby facilitating tissue repair and regeneration. One significant advancement is the development of electrospun scaffolds with nanoscale fibers that replicate the intricate fibrous architecture of natural tissues. This promotes more effective cell–material interactions and improves the integration of implanted scaffolds with host tissue. In recent years, the functionality of electrospun scaffolds has been significantly enhanced by the incorporation of bioactive molecules, such as growth factors, nanoparticles, and drugs. These composite scaffolds are engineered to deliver therapeutic agents in a controlled manner, providing a localized and sustained release that can stimulate cellular activities and enhance tissue healing. In this regard, the current study on the delivery of FUC from electrospun PVA/HL nanofibers highlights several biomimetic aspects that are crucial for advancing wound care technologies. PVA and HL are selected for their biocompatibility and structural similarities to natural ECM components, which mimic the native tissue environment. By combining the natural properties of FUC and HL with the structural advantages of electrospun PVA nanofibrous mats, this study aims to replicate the controlled release and localized delivery mechanisms found in biological systems. Thus, this work aligns well with biomimetic principles to create advanced, biocompatible delivery systems for various medical applications, including wound dressings.

## 2. Materials and Methods

### 2.1. Materials

PVA (MW = 89.000–98.000, 99+% hydrolyzed) and glutaraldehyde (Glutaraldehyde 50%, Pentanedial, MW: 100.12 g/mol) were procured from Sigma Aldrich (St. Louis, MO, USA). HL was fabricated by the halophilic bacterium of H. smyrnensis, isolated from the Çamaltı Saltern area, and microbial fermentation. FUC (80 kD from Fucus vesiculosus) was obtained from the Pharma Assist Company (Tipperary, Ireland).

### 2.2. Halomonas Levan’s Production and Purification

HL was produced under controlled bioreactor conditions with a working volume of 100 mL in a 500 mL Erlenmeyer flask, under 37 °C and 180 rpm. The pH was adjusted to 7 by adding 1 M of NaOH or HCl to the growth medium. HL was isolated by the centrifugation of growth medium at 10,000 rpm for 20 min. The supernatant was treated with an equal volume of ethanol and kept at 18 °C overnight after stirring. The precipitate of the ethanol dispersion was centrifuged at 12,000 rpm for 30 min, and the supernatant was re-dissolved in distilled water, dialyzed in several runs of distilled water for three days, and then lyophilized.

### 2.3. Preparation of Electrospun Fibrous Mats

To create the mats, 12% (*w*:*v*) PVA was dissolved in 20 mL of distilled water at 120 °C and 250 rpm for 45 min. After cooling down, 1% (*w*:*v*) HL was added and stirred for 45 min at 250 rpm. FUC was used as an antibiotic agent at 0.5, 0.65, and 0.75 mg/mL concentrations (Table 1). The solution was transferred into a pump and electrospinning was carried out for 3 h with a 0.6 mL/h flow rate, 25 kV applied voltage, and 15 cm needle–collector distance.

### 2.4. Morphological Characterization

A SEM analysis was employed to scrutinize the structure and surface attributes of the electrospun nanofibers. The Evo LS 10 model from ZEISS was used for these investigations. To evaluate the distribution of electrospun nanofiber diameters within the tissue scaffold, an average of 100 random readings were taken for each sample. The measurements were performed using ImageJ software (v1.8.0) from Olympus AnalySIS, Waltham, MA, USA.

### 2.5. Fourier-Transform Infrared Spectroscopy (FTIR) Analysis

The chemical composition of the samples was analyzed using a FTIR spectrophotometer (JASCO FT/IR-4700, Tokyo, Japan), operating in transmission mode with a resolution of 4 cm^−1^ (32 scans), across the 4000–400 cm^−1^ range.

### 2.6. Differential Scanning Calorimetry

The thermal properties of the nanofibers were tested with a differential scanning calorimetry (DSC) device (Shimadzu, Tokyo, Japan), which was set to a speed of 5 °C/min and a temperature in the range of 25–400 °C.

### 2.7. Tensile Testing

Tensile strength assessments were conducted on individual samples using a Shimadzu device (EZ-X, Tokyo, Japan), which operated at a speed of 5 mm/min under a force of 0.1 N. Before testing, the electrospun nanofibers were fashioned into specimens measuring 10 mm in width and 50 mm in length. The thickness of each sample was determined using a digital micrometer (Mitutoyo MTI Corp., Auburn, WA, USA) and the measurements were input into the system for documentation.

### 2.8. Biological Characterization and Cell Viability Tests

All mats were meticulously sectioned into squares of 1 cm × 1 cm and subsequently placed into 24-well cell culture plates for biological characterization. Each sample underwent a series of sterilization procedures using an alcohol gradient, followed by a series of five PBS rinses. To ensure stability, the samples were immersed in cell culture medium at a temperature of 37 °C overnight before starting the process of cell seeding. The mouse fibroblast cell line L929 was then introduced onto the mats at a density of 2 × 10^4^ cells per well for each sample. The cultures were then maintained at a temperature of 37 °C within a humidified air environment of 5% CO_2_ in an incubator.

The metabolic activity of the cells on the fibrous samples was assessed using the WST1 assay kit (Roche Applied Science, Penzberg, Germany) over a period of 24, 48, and 72 h. A control group was established by introducing cells into tissue culture plates devoid of any scaffold.

The cell viability was calculated as a percentage of the cell viability in comparison to the control group, with the control group being considered 100% viable. The biocompatibility of the cells was assessed using 4′,6-diamidino-2-phenylindole (DAPI) staining on cells seeded onto fibrous samples created via the electrospinning technique.

All biological analyses, including the examination of cell metabolic activity using the WST1 assay kit and the evaluation of cell biocompatibility through DAPI staining, were conducted in triplicate for each sample.

### 2.9. In Vitro Drug Release Tests

Firstly, five different concentrations of FUC (0.2, 0.4, 0.6, 0.8, and 1 μg/mL) were used to establish the linear calibration curves of the pristine FUC. Subsequently, the FUC release characteristics from the nanofibers were examined at various time intervals. The first step was to weigh and place 10, 13, and 15 mg of FUC-loaded nanofibers into Eppendorf tubes with 1 mL of Phosphate-Buffered Saline (PBS) (pH: 7.4). FUC’s release from the PVA-HL nanofibers was carried out using a thermal shaker (BIOSAN TS-100C, Riga, Latvia). The test was conducted using 1 mL of fresh PBS. At various time intervals, the 1 mL of PBS was removed from the Eppendorf tubes and added to a quartz bath with a 1 mL volume capacity. The FUC’s release from the nanofibers was tracked using UV spectrophotometry in a PBS environment, with measurements taken at a wavelength of 214 nm. The process begins by incubating the PBS solution and samples in the Eppendorf tubes in the thermo-shaker for 15 min. Next, the solutions were removed from the Eppendorf tubes and analyzed with a spectrophotometer. After analysis, fresh PBS was added, and the samples were kept in the thermo-shaker for 120 min.

### 2.10. Statistical Analysis

All experiments were carried out in triplicate to ensure statistical significance. Statistical analysis was performed using the unpaired Student’s *t*-test, with differences considered significant at *p* < 0.05.

## 3. Results and Discussion

### 3.1. SEM Analysis

**Figure 1:**
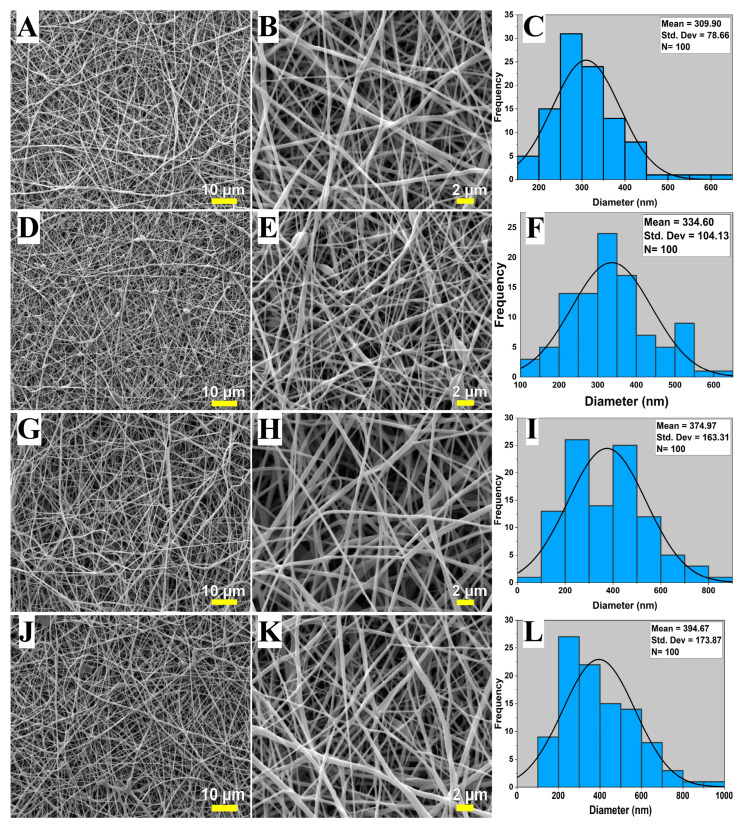
Scanning electron microscopy images and the corresponding distribution of fiber diameters of electrospun nanofibrous mats: PVA-HL (**A**–**C**), PVA-HL-10FUC (**D**–**F**), PVA-HL-13FUC (**G**–**I**), and PVA-HL-15FUC (**J**–**L**).

Electrospun mats were analyzed using SEM to identify their surface morphologies. SEM images, along with histograms of fiber diameters, are given in Figure 1. The electrospun mats, both control and drug-loaded, display a relatively consistent nanofiber structure, which was also shown in previous studies [31,32,33]. However, the presence of bead-like imperfections was also evidenced in the nanofiber mats presented in Figure 1B. The mean diameter of PVA-HL, PVA-HL-10FUC, PVA-HL-13FUC, and PVA-HL-15FUC is 310 ± 79, 335 ± 104, 375 ± 163, and 395 ± 174 nm, respectively. An increase in the diameter of FUC-loaded electrospun fibers compared to the control electrospun fibers was observed. Prior investigations have substantiated a phenomenon wherein nanofiber diameter augmentation occurs upon drug loading [34]. Another study conducted with PVA/Chitosan/FUC found similar results, showing that as the FUC amount increased, the diameter of the nanofibers also increased. The increase in the average diameter of nanofibers with a higher FUC content can be attributed to changes in solution viscosity at varying concentrations. It was shown that elevated solution viscosity results in greater polymer chain entanglement and increased resistance to stretching [35]. As a result, higher concentrations of FUC result in nanofibers with larger diameters.

### 3.2. FTIR Analysis

**Figure 2:**
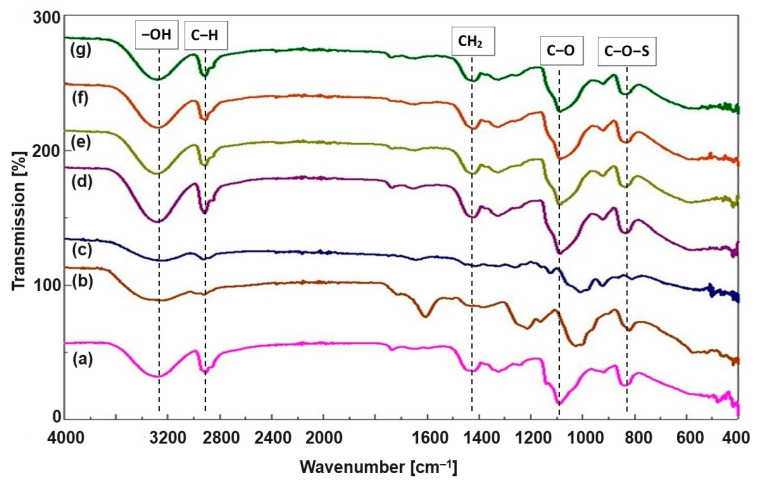
FTIR spectra of electrospun nanofibrous mats: PVA (**a**), FUC (**b**), HL (**c**), PVA-HL (**d**), PVA-HL-10FUC (**e**), PVA-HL-13FUC (**f**), and PVA-HL-15FUC (**g**).

The FTIR spectra of the fabricated electrospun mats based on PVA, HL, and FUC, along with the spectra of PVA-HL, HL, and FUC, are shown in Figure 2. Pristine PVA nanofibers exhibited transmittance peaks at ~820 and ~2875 cm^−1^ [36], which were attributed to C–O–S and C–H groups, respectively. An important peak verified at ~1100 cm^−1^ is attributed to PVA crystallization and is related to a –COOH stretching vibration [32]. A large number of –OH groups are present in PVA, and, similarly, HL also has an excess of –OH groups. The broad peak at 3300–3200 cm^−1^ corresponds to the stretching of –OH groups. The C–O peak was observed in the 1300–1000 cm^−1^ region. A homogenous amalgam can be formed due to the formation of hydrogen bonds because of these –OH groups [37]. The decrease in intensity of the –OH group peak is the confirmation of PVA-HL crosslinking. The peak at 2910 cm^−1^ is assigned to the C–H stretching of the alkyl chain [38]. The peak at 1420 cm^−1^ is characteristic of CH_2_ bending. Similarly, in the FTIR spectra of HL, the broad absorption peak in the 3500–3200 cm^−1^ region is due to–OH stretching. Its –C–O–C– symmetric vibration also presents some characteristic bands at 950, 1000, and 1100 cm^−1^ due to its fructose ring and glycosidic linkage [17,39]. In the FTIR spectrum of FUC, the characteristic peak in the range of 1260–1160 cm^−1^ is because of the asymmetric stretching of S=O and the peak at 845 cm^−1^ is due to the C–O–S stretching of the sulfate group [40]. The strong and broad band in the region of 3400–3200 cm^−1^ is due to the –OH stretching of polyphenolic alcohols [41]. In the FTIR spectra of PVA-HL-FUC, similar peaks as that of PVA-HL can be seen; for example, there is a broad peak at 3300–3200 cm^−1^ which agrees with –OH functional group stretching, but in these PVA-HL-FUC mats it can be seen that the intensity of this peak is lowered, which is the sign of the crosslinking of FUC with PVA-HL. The C–O peak in the 1300–1000 cm^−1^ region is also affected in intensity because of the incorporation of FUC. Therefore, it is concluded that PVA-HL-FUC mats have successfully been synthesized.

### 3.3. Differential Scanning Calorimetry Analysis

**Figure 3:**
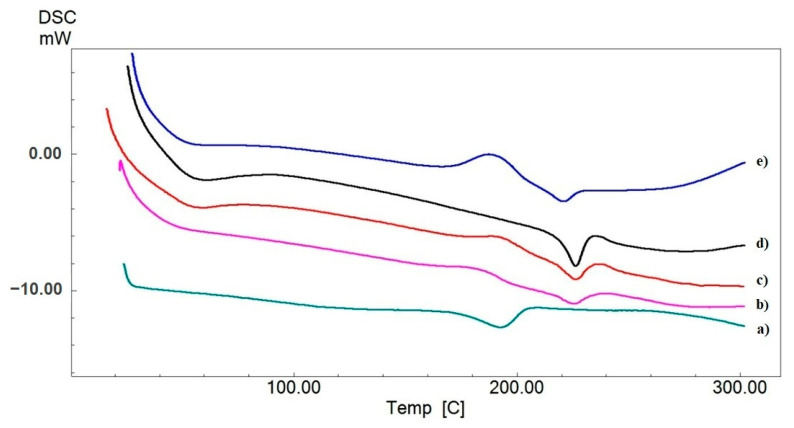
DSC graph of electrospun nanofibrous mats: PVA (**a**), PVA-HL (**b**), PVA-HL-10FUC (**c**), PVA-HL-13FUC (**d**), and PVA-HL-15FUC (**e**).

Thermal characterization techniques can be very helpful in determining many physical and chemical changes like the phase transition, glass transition, and melting temperature of molecular structures [42]. The DSC curves of electrospun mats of PVA, PVA-HL, PVA-HL-10FUC, PVA-HL-13FUC, and PVA-HL-15FUC are shown in Figure 3. The PVA polymer is partially crystalline at both its glass transition temperature (i.e., specific for the amorphous phase) and melting isotherm, which specifically refers to the crystalline phase [43]. The peak observed at 222 °C in the thermogram obtained for PVA-HL corresponds to the melting temperature of PVA. The glass transition temperature is observed at a lower temperature than that of PVA and at a higher temperature than HL, i.e., at 42 °C. It was observed that the addition of HL to PVA resulted in a shift in the glass transition temperature of PVA. The glass transition temperatures of PVA-HL-10FUC, PVA-HL-13FUC, and PVA-HL-15FUC are 60 °C, 57 °C, and 55 °C, respectively. This shift is mainly because of the interaction of the antibiotic agent FUC with the polymer. The melting points of PVA-HL-10FUC, PVA-HL-13FUC, and PVA-HL-15FUC were measured to be 224 °C, 226 °C, and 220 °C, respectively. A possible explanation for the initial increase in the melting point could be attributed to the strengthening of molecular interactions and increased crystallinity at lower FUC concentrations. Conversely, the observed decrease at higher FUC concentrations is likely due to phase separation, enhanced amorphous characteristics, or plasticization effects.

### 3.4. Tensile Strength

**Figure 4:**
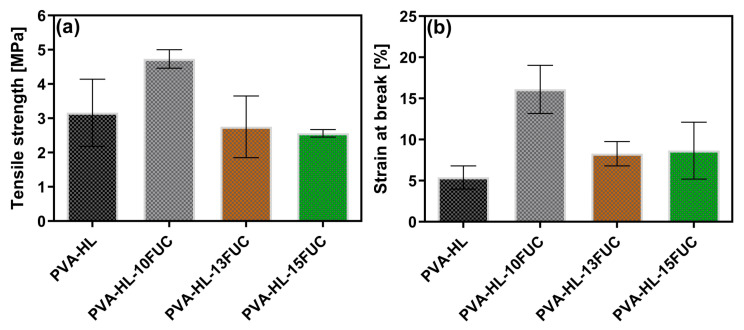
Tensile test measurement of nanofibrous electrospun mats: tensile strength (**a**) and strain at break (**b**).

The mechanical properties of electrospun mats for wound dressings are very important because they are used close to the skin surface. These wound dressings should not tear easily when stretched [44]. Therefore, to test this hypothesis, tensile testing was conducted on these electrospun mats. The tensile behaviors of electrospun mats are affected by the chemical composition of the materials used [45]. Both the tensile strength and strain were investigated at the break of electrospun nanofiber mats and the results are shown in Figure 4. The control PVA-HL electrospun mat showed a tensile strength of 3.14 ± 0.98 MPa (Figure 4a) and a strain at a break of 5.40 ± 1.41% (Figure 4b). When FUC was loaded into these electrospun nanofibers at different concentrations, the tensile strength values were 4.73 ± 0.27 MPa, 2.75 ± 0.90 MPa, and 2.56 ± 0.11 MPa for PVA-HL-10FUC, PVA-HL-13FUC, and PVA-HL-15FUC, respectively (Figure 4a). The inferred values of the strain at break for PVA-HL-10FUC, PVA-HL-13FUC, and PVA-HL-15FUC were 16.11 ± 2.93%, 8.28 ± 1.48%, and 8.65 ± 3.46%, respectively (Figure 4b). It can be seen that the loading of FUC results in an increase in tensile strength for PVA-HL-10FUC compared to PVA-HL. It was reported that loading drugs could increase the tensile strength of nanofibers [46]. For samples PVA-HL-13FUC and PVA-HL-15FUC, the value of their tensile strength decreased compared to the control PVA-HL with the addition of FUC. Hence, it can be concluded that a higher ratio of PVA and HL in the mats results in greater tensile strength. The same behavior is observed for the strain at break; when PVA and HL concentrations are higher, the elongation rate improves. In a study conducted by Ayran et al., it was found that a high amount of FUC led to a decrease in tensile strength properties [28]. The findings from this study and ours support the idea that FUC has a favorable effect on mechanical strength at lower concentrations. It is important to mention that the reported values of tensile strength in the case of human skin vary within the range of 1–32 MPa, whilst its strain at break ranges from 17 to 207%. However, due to the heterogeneity of human skin, these values depend on several factors such as age, skin color, and genetic heritage [47,48]. Therefore, one can consider the mechanical properties of our fabricated nanofibrous mats to be within an acceptable range for wound dressing applications.

### 3.5. Cell Viability

**Figure 5:**
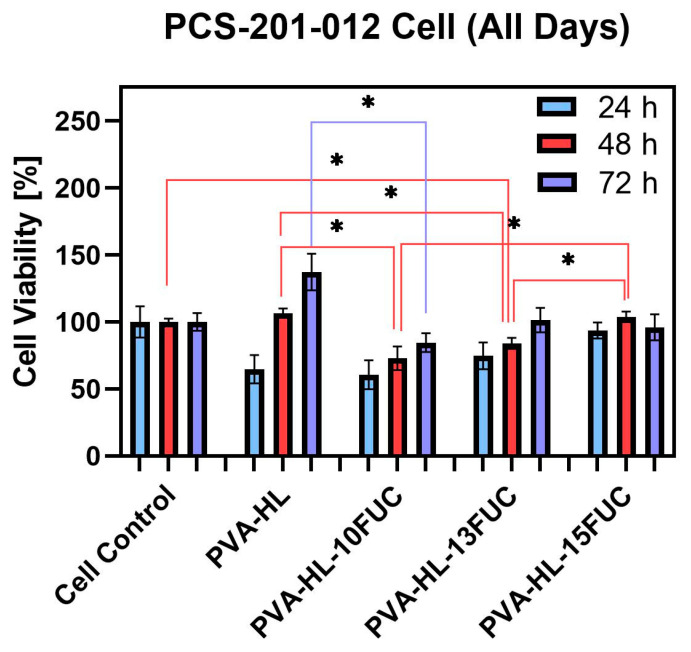
Cell viability of control and electrospun nanofibrous mats at 24, 48, and 72 h (* *p* ≤ 0.5; data calculated as mean ± SD, n = 3).

The cell viability of the electrospun nanofibers of PVA-HL and PVA-HL-FUC at different concentrations strongly depends on their chemical composition and the effect of the environment on the material. Cell viability refers to the ability of cells to remain healthy and functional in an environment, which determines the suitability of materials for biological applications. In this study, the CCK-8 cell proliferation assay was used to determine cell viability. After sterilization, the samples were placed in cell culture medium, and extracts were collected at 24, 48, and 72 h and then applied to pre-cultured cells. Figure 5 shows that the control sample (i.e., PVA-HL), PVA-HL-10FUC, and PVA-HL-13FUC exhibited no cytotoxicity up to 72 h, as cell viability increased over time. However, in the PVA-HL-15FUC sample, cell viability slightly decreased after 48 h. This reduction in cell proliferation may be due to rapid cell proliferation after 48 h, leading to crowding in the growth area, which consequently caused a decline in cell viability at 72 h [49]. Ilhan et al. reported that the decrease in cell viability could be attributed to increased cell confluency and detachment from the monolayer [50].

It is important to note that the differences observed when comparing all sample groups were statistically significant (Figure 5). Their corresponding *p* values, as indicated in Figure 5, are summarized in Table 2 for easier visualization.

### 3.6. Release Behavior of the FUC from the PVA-HL Nanofibers

**Figure 6:**
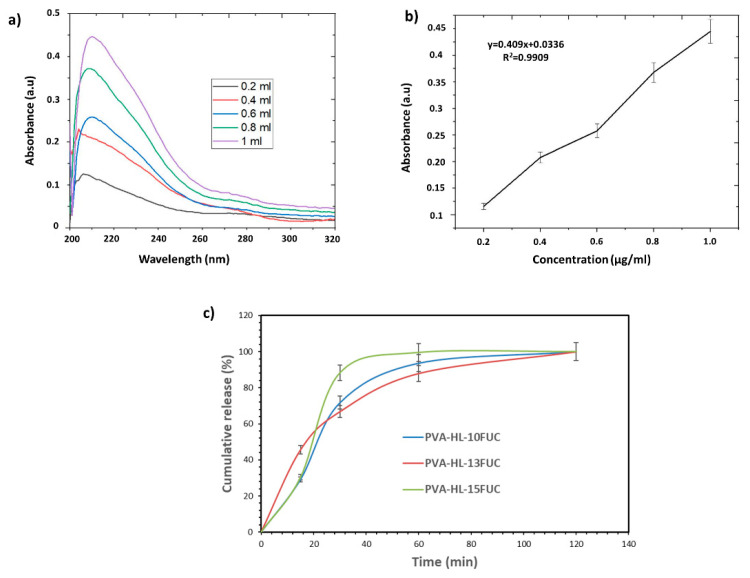
The linear calibration curve (**a**) and absorbance graph of FUC (**b**). The cumulative release behavior of FUC from the PVA-HL nanofibers (**c**).

Figure 6a shows the linear calibration curve for FUC, while Figure 6b presents the absorbance graph obtained from the calibration curve at 214 nm. One can observe in Figure 6c that the release curve exhibits a typical drug release pattern. After 2 h, it was observed that all FUC had been released from each nanofiber. Monitoring the release of FUC is crucial, as it is water-soluble and impacts the cellular activity of nanofibers. Therefore, an important criterion in evaluating nanofibers is the amount of FUC released. It was discovered that the release rate of FUC from the PVA-HL-15FUC nanofiber was faster than that of other nanofibers loaded with FUC. Only 88% of the FUC was released from the PVA-HL-15FUC nanofiber after 30 min. In this research, PVA was used to cause a burst release of the drugs, achieving the goal of rapid PVA degradation and drug release for wound dressing applications [51].

## 4. Conclusions

We report, in this study, on the successful synthesis and characterization of polyvinyl alcohol (PVA)/Halomonas Levan (HL) electrospun nanofiber mats, into which various concentrations of Fucoidan (FUC) were incorporated. Following SEM investigations, it was observed that the nanoscale fibers exhibited a slight presence of beads only at the lowest FUC concentration, while the other fibers were bead-free, continuous, and smoothly structured. Additionally, an increase in fiber diameter was noted with FUC loading. FTIR analysis revealed the presence of characteristic functional groups and confirmed a successful crosslinking between PVA and HL, as well as the incorporation of FUC. The DSC analysis provided insights into the thermal behavior of the mats, revealing shifts in the glass transition, and melting temperatures due to interactions between the components. The results of mechanical testing indicated that the incorporation of FUC generally decreased the values of tensile strength. Cell viability assays demonstrated the non-cytotoxic behavior of most formulations. However, a decrease in viability was observed for the highest FUC concentration after 48 h, most likely due to increased cell confluency.

This summary of the findings suggests that the developed electrospun nanofibrous mats possess promising characteristics for wound dressing applications, including appropriate mechanical properties, morphology, and biocompatibility. Further optimization of the FUC concentration, along with additional in vitro and in vivo studies, is needed to fully evaluate the efficacy and potential clinical relevance of these mats for wound healing therapies. Moreover, it is important to emphasize that the fusion of biomimetic design principles with electrospinning technology is currently driving significant progress in the biomedical field. These advancements are leading to the creation of novel materials and devices that not only mimic the natural tissue environment but also enhance the body’s intrinsic healing capabilities. As research in this field continues to evolve, one can anticipate further breakthroughs that will enhance patient outcomes and expand the possibilities of regenerative medicine and beyond.

## Figures and Tables

**Table 1:** Different Fucoidan (FUC) quantities used to prepare the solutions investigated in this work.

Solution Codes	PVA	HL	FUC	Distilled Water
PVA-HL	2.4 g	-	-	20 mL
PVA-HL-10FUC	2.4 g	24 mg	10 mg	20 mL
PVA-HL-13FUC	2.4 g	24 mg	13 mg	20 mL
PVA-HL-15FUC	2.4 g	24 mg	15 mg	20 mL

**Table 2:** Statistically significant differences observed at 48 h and 72 h when comparing all sample groups.

Comparison	Time Interval (hours)	*p*-Value
Cell control vs. PVA-HL-13FUC	48	0.03
PVA-HL vs. PVA-HL-10FUC	48	0.04
PVA-HL vs. PVA-HL-10FUC	72	0.04
PVA-HL vs. PVA-HL-13FUC	48	0.03
PVA-HL-10FUC vs. PVA-HL-15FUC	48	0.05
PVA-HL-13FUC vs. PVA-HL-15FUC	48	0.02

## Data Availability

The original contributions presented in the study are included in the article, further inquiries can be directed to the corresponding authors.
